# 4-Ferrocenyl-1-methyl-3-benzoyl­spiro­[pyrrolidine-2,11′-indeno­[1,2-*b*]quinoxaline]

**DOI:** 10.1107/S1600536812048349

**Published:** 2012-11-30

**Authors:** B. Vijayakumar, A. R. Sureshbabu, D. Gavaskar, R. Raghunathan, D. Velmurugan

**Affiliations:** a Centre of Advanced Study in Crystallography and Biophysics, University of Madras, Maraimalai (Guindy) Campus, Chennai 600 025, India; b Department of Organic Chemistry, University of Madras, Maraimalai (Guindy) Campus, Chennai 600 025, India

## Abstract

In the title compound, [Fe(C_5_H_5_)(C_31_H_24_N_3_O)], the pyrrolidine ring adopts a twist conformation. The pyrrolidine ring is almost perpendicular to the indeno­quinoxaline ring system, making a dihedral angle of 84.44 (5)°. The cyclo­penta­dienyl rings of the ferrocene moiety adopt an eclipsed conformation. The crystal packing features weak C—H⋯N and C—H⋯π inter­actions.

## Related literature

For the biological activity of ferrocene derivatives, see: Jaouen *et al.* (2004 ▶); Biot *et al.* (2004 ▶); Fouda *et al.* (2007 ▶). For related structures, see: Kamala *et al.* (2009 ▶); Gunasekaran *et al.* (2010 ▶); Vijayakumar *et al.* (2012 ▶); For puckering and asymmetry parameters, see: Cremer & Pople (1975 ▶); Nardelli (1983 ▶).
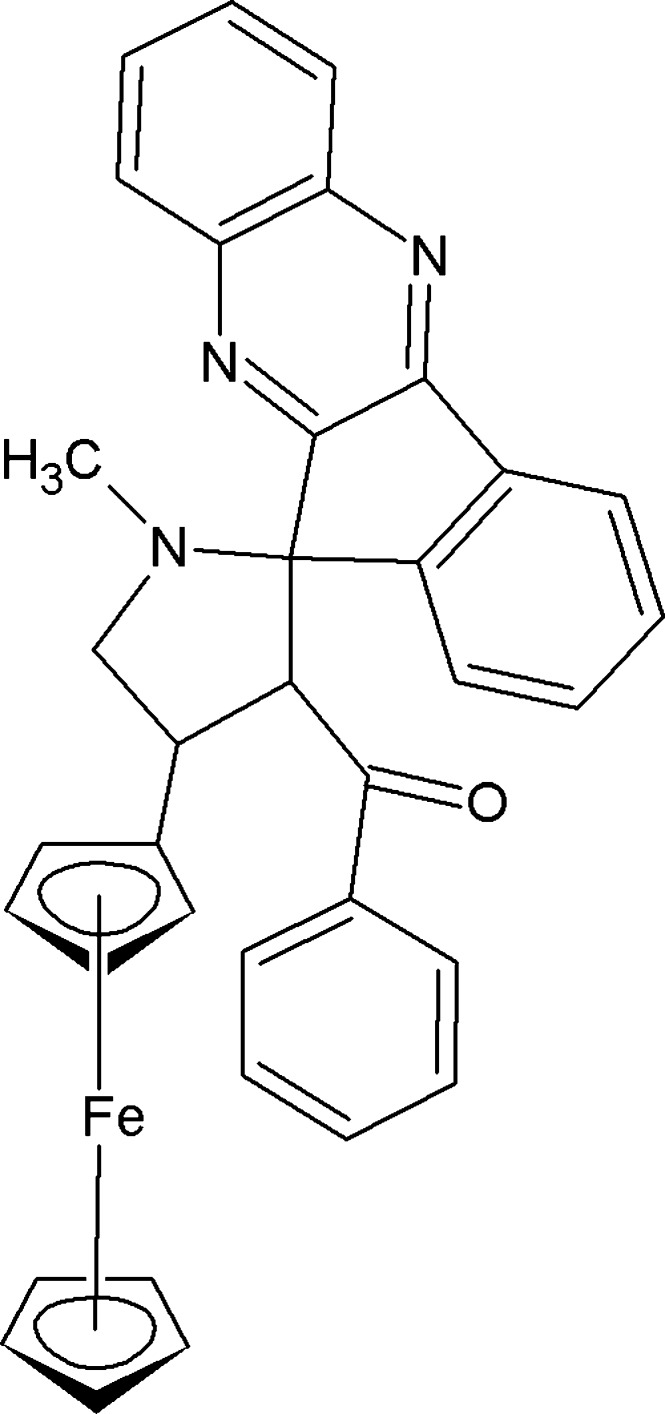


## Experimental

### 

#### Crystal data

[Fe(C_5_H_5_)(C_31_H_24_N_3_O)]
*M* *_r_* = 575.47
Monoclinic, 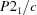

*a* = 10.8091 (18) Å
*b* = 12.326 (2) Å
*c* = 20.989 (3) Å
β = 100.654 (8)°
*V* = 2748.2 (8) Å^3^
*Z* = 4
Mo *K*α radiation
μ = 0.58 mm^−1^
*T* = 293 K
0.2 × 0.2 × 0.2 mm

#### Data collection

Bruker SMART APEXII area-detector diffractometer
Absorption correction: multi-scan (*SADABS*; Bruker, 2008 ▶) *T*_min_ = 0.890, *T*_max_ = 0.890
27206 measured reflections
6927 independent reflections
5624 reflections with *I* > 2σ(*I*)
*R*_int_ = 0.028

#### Refinement

*R*[*F* ^2^ > 2σ(*F* ^2^)] = 0.036
*wR*(*F* ^2^) = 0.106
*S* = 1.01
6927 reflections
371 parameters
H-atom parameters constrained
Δρ_max_ = 0.30 e Å^−3^
Δρ_min_ = −0.44 e Å^−3^

### 

Data collection: *APEX2* (Bruker, 2008 ▶); cell refinement: *SAINT* (Bruker, 2008 ▶); data reduction: *SAINT*; program(s) used to solve structure: *SHELXS97* (Sheldrick, 2008 ▶); program(s) used to refine structure: *SHELXL97* (Sheldrick, 2008 ▶); molecular graphics: *ORTEP-3* (Farrugia, 2012 ▶); software used to prepare material for publication: *SHELXL97* and *PLATON* (Spek, 2009 ▶).

## Supplementary Material

Crystal structure: contains datablock(s) global, I. DOI: 10.1107/S1600536812048349/bt6861sup1.cif

Structure factors: contains datablock(s) I. DOI: 10.1107/S1600536812048349/bt6861Isup2.hkl

Supplementary material file. DOI: 10.1107/S1600536812048349/bt6861Isup3.mol

Additional supplementary materials:  crystallographic information; 3D view; checkCIF report

## Figures and Tables

**Table 1:** Hydrogen-bond geometry (Å, °) *Cg*3 is the centroid of the C20–C24 ring.

*D*—H⋯*A*	*D*—H	H⋯*A*	*D*⋯*A*	*D*—H⋯*A*
C25—H25⋯N3	0.93	2.55	3.351 (2)	144
C35—H35⋯*Cg*3^i^	0.93	2.83	3.616 (2)	143

Symmetry code: (i) 
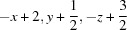
.

**Fig. 1.**
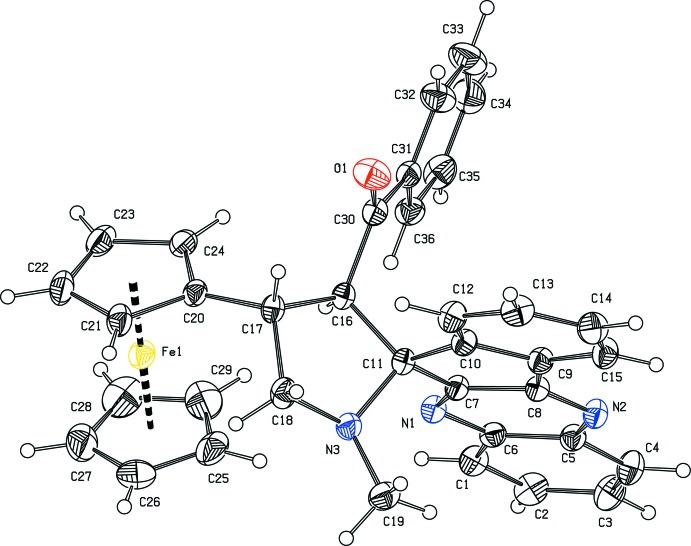
Molecular structure of the title compound, showing displacement ellipsoids drawn at the 30% probability level. H atoms are presented as a small spheres of arbitrary radius.

**Fig. 2.**
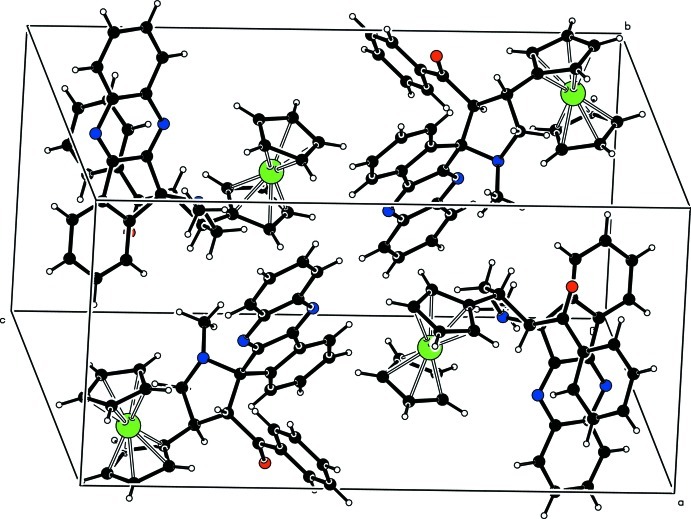
The molecular packing viewed down the a axis.

## supplementary crystallographic information

### Comment

Ferrocene attached compounds are well known to have biological activities like antimalarial, antifungal (Biot *et al.*, 2004), antitumor (Jaouen *et al.*, 2004), and antibacterial (Fouda *et al.*, 2007). Against this background, the information of molecular conformations and crystal packing of title compound was obtained and analyzed using X-ray diffraction study. The bond lengths and angles of titled compound agree with those observed in other ferrocene derivative 3'-Ferrocenylcarbonyl-1'-methyl-4'- phenylspiro[indeno[2,3-b]quinoxaline-11,2'-pyrrolidine] (Vijayakumar *et al.* 2012).

The pyrrolidine ring adopts a twisted conformation with the puckering parameters q_2_ and φ and the smallest displacement asymmetric parameters, δ, as follows: q2 = 0.399 (1) A, φ = 26.0 (2)° and Δ_2_(C17) =7.29 (14)°. The dihedral angle between the pyrrolidine ring and indeno-quinoxaline ring system is 84.44 (5)° which clearly shows that the both rings are almost perpendicular to each other. The Cg3 (C20—C24) and Cg4 (C25—C29) are the centroids cyclopentadinene rings where the Fe1-Cg3 and Fe1-Cg4 distances are 1.6466 (8) and 1.6512 (10) Å, respectively and the Cg3-Fe1-Cg4 angle is 179.40 (5)°. In addition to the van der Waals interactions, the crystal packing (Fig.2) is stabilized by weak C—H···N and C—H···π interactions (Table 1).

### Experimental

Ninhydrin (1 mM) and 1, 2-phenylenediamine (1 mM) were mixed and stirred with 10mL of methanol for 10 min. To this mixture 1 mM of Sarcosine and 1 mM of ferrocene derived dipolarophile were added and was refluxed up to the end of the reaction as observed by TLC. The solvent content from the mixture was removed under reduced pressure and the crude product was obtained. Using column chromatography the crude extract was purified by 4:1 ratio of petroleum ether and ethyl acetate. Finally, single crystals suitable for the X-ray diffraction were obtained by slow evaporation at room temperature.

### Refinement

Hydrogen atoms were placed in calculated positions with C—H ranging from 0.93Å to 0.98Å and refined using the riding model approximation with a fixed isotropic displacement parameter of *U*_iso_(H) = 1.2 *U*_eq_(C).

### Crystal data

**Table:**

[Fe(C_5_H_5_)(C_31_H_24_N_3_O)]	*Z* = 4
*M**_r_* = 575.47	*F*(000) = 1200
Monoclinic, *P*2_1_/*c*	*D*_x_ = 1.391 Mg m^−^^3^
Hall symbol: -P 2ybc	Mo *K*α radiation, λ = 0.71073 Å
*a* = 10.8091 (18) Å	θ = 2.0–28.3°
*b* = 12.326 (2) Å	µ = 0.58 mm^−^^1^
*c* = 20.989 (3) Å	*T* = 293 K
β = 100.654 (8)°	Block, brown
*V* = 2748.2 (8) Å^3^	0.2 × 0.2 × 0.2 mm

### Data collection

**Table:**

Bruker SMART APEXII area-detector diffractometer	6927 independent reflections
Radiation source: fine-focus sealed tube	5624 reflections with *I* > 2σ(*I*)
Graphite monochromator	*R*_int_ = 0.028
ω and φ scans	θ_max_ = 28.6°, θ_min_ = 1.9°
Absorption correction: multi-scan (*SADABS*; Bruker, 2008)	*h* = −14→14
*T*_min_ = 0.890, *T*_max_ = 0.890	*k* = −16→14
27206 measured reflections	*l* = −28→27

### Refinement

**Table:**

Refinement on *F*^2^	Primary atom site location: structure-invariant direct methods
Least-squares matrix: full	Secondary atom site location: difference Fourier map
*R*[*F*^2^ > 2σ(*F*^2^)] = 0.036	Hydrogen site location: inferred from neighbouring sites
*wR*(*F*^2^) = 0.106	H-atom parameters constrained
*S* = 1.01	*w* = 1/[σ^2^(*F*_o_^2^) + (0.0582*P*)^2^ + 0.6864*P*] where *P* = (*F*_o_^2^ + 2*F*_c_^2^)/3
6927 reflections	(Δ/σ)_max_ = 0.002
371 parameters	Δρ_max_ = 0.30 e Å^−^^3^
0 restraints	Δρ_min_ = −0.44 e Å^−^^3^

### Special details

**Table:**

Geometry. All esds (except the esd in the dihedral angle between two l.s. planes) are estimated using the full covariance matrix. The cell esds are taken into account individually in the estimation of esds in distances, angles and torsion angles; correlations between esds in cell parameters are only used when they are defined by crystal symmetry. An approximate (isotropic) treatment of cell esds is used for estimating esds involving l.s. planes.
Refinement. Refinement of F^2^ against ALL reflections. The weighted R-factor wR and goodness of fit S are based on F^2^, conventional R-factors R are based on F, with F set to zero for negative F^2^. The threshold expression of F^2^ > 2sigma(F^2^) is used only for calculating R-factors(gt) etc. and is not relevant to the choice of reflections for refinement. R-factors based on F^2^ are statistically about twice as large as those based on F, and R- factors based on ALL data will be even larger.

### Fractional atomic coordinates and isotropic or equivalent isotropic displacement parameters (Å^2^)

**Table:**

	*x*	*y*	*z*	*U*_iso_*/*U*_eq_	
C1	0.79681 (14)	0.51846 (13)	0.70367 (8)	0.0417 (3)	
H1	0.8397	0.5059	0.7457	0.050*	
C2	0.80655 (16)	0.61717 (14)	0.67526 (9)	0.0480 (4)	
H2	0.8561	0.6713	0.6980	0.058*	
C3	0.74260 (17)	0.63721 (15)	0.61233 (9)	0.0513 (4)	
H3	0.7494	0.7048	0.5936	0.062*	
C4	0.67032 (16)	0.55878 (14)	0.57805 (9)	0.0475 (4)	
H4	0.6284	0.5733	0.5361	0.057*	
C5	0.65853 (13)	0.45581 (12)	0.60556 (7)	0.0351 (3)	
C6	0.72248 (13)	0.43585 (12)	0.66985 (7)	0.0332 (3)	
C7	0.63802 (12)	0.26813 (11)	0.66576 (6)	0.0314 (3)	
C8	0.57653 (13)	0.28695 (12)	0.60063 (6)	0.0323 (3)	
C9	0.50426 (13)	0.19007 (12)	0.57774 (7)	0.0345 (3)	
C10	0.52049 (13)	0.11230 (12)	0.62698 (7)	0.0339 (3)	
C11	0.60275 (13)	0.15690 (11)	0.68865 (6)	0.0314 (3)	
C12	0.45706 (15)	0.01405 (13)	0.61673 (8)	0.0422 (3)	
H12	0.4672	−0.0388	0.6489	0.051*	
C13	0.37828 (15)	−0.00376 (14)	0.55751 (8)	0.0459 (4)	
H13	0.3344	−0.0688	0.5504	0.055*	
C14	0.36392 (15)	0.07330 (15)	0.50904 (8)	0.0469 (4)	
H14	0.3112	0.0593	0.4696	0.056*	
C15	0.42706 (15)	0.17096 (14)	0.51848 (7)	0.0424 (3)	
H15	0.4179	0.2228	0.4857	0.051*	
C16	0.71773 (12)	0.08849 (11)	0.72140 (6)	0.0306 (3)	
H16	0.7793	0.1376	0.7466	0.037*	
C17	0.66473 (13)	0.01436 (12)	0.76915 (7)	0.0334 (3)	
H17	0.6455	−0.0563	0.7483	0.040*	
C18	0.53991 (14)	0.06744 (14)	0.77724 (8)	0.0414 (3)	
H18A	0.4690	0.0226	0.7580	0.050*	
H18B	0.5373	0.0783	0.8227	0.050*	
C19	0.41254 (17)	0.22002 (16)	0.72858 (9)	0.0522 (4)	
H19A	0.3545	0.1694	0.7045	0.078*	
H19B	0.4159	0.2844	0.7032	0.078*	
H19C	0.3850	0.2386	0.7681	0.078*	
C20	0.75808 (14)	−0.00309 (11)	0.83087 (7)	0.0340 (3)	
C21	0.73116 (17)	−0.03810 (13)	0.89135 (7)	0.0433 (4)	
H21	0.6516	−0.0514	0.9005	0.052*	
C22	0.84766 (19)	−0.04913 (14)	0.93543 (8)	0.0506 (4)	
H22	0.8571	−0.0704	0.9786	0.061*	
C23	0.94568 (17)	−0.02255 (14)	0.90278 (8)	0.0499 (4)	
H23	1.0312	−0.0233	0.9204	0.060*	
C24	0.89125 (15)	0.00566 (13)	0.83792 (8)	0.0404 (3)	
H24	0.9351	0.0262	0.8057	0.048*	
C25	0.7390 (2)	0.25002 (16)	0.87637 (10)	0.0648 (6)	
H25	0.6786	0.2628	0.8394	0.078*	
C26	0.7154 (2)	0.21523 (17)	0.93713 (11)	0.0692 (6)	
H26	0.6371	0.2004	0.9474	0.083*	
C27	0.8317 (3)	0.20723 (18)	0.97903 (10)	0.0738 (7)	
H27	0.8439	0.1866	1.0223	0.089*	
C28	0.9260 (3)	0.23524 (18)	0.94526 (13)	0.0755 (7)	
H28	1.0119	0.2362	0.9619	0.091*	
C29	0.8690 (3)	0.26178 (16)	0.88154 (12)	0.0723 (6)	
H29	0.9105	0.2834	0.8486	0.087*	
C30	0.78362 (13)	0.02473 (12)	0.67524 (7)	0.0352 (3)	
C31	0.89465 (13)	0.07269 (13)	0.65201 (7)	0.0359 (3)	
C32	0.95018 (16)	0.01150 (17)	0.60885 (9)	0.0523 (4)	
H32	0.9162	−0.0554	0.5946	0.063*	
C33	1.05538 (18)	0.0497 (2)	0.58717 (10)	0.0638 (5)	
H33	1.0920	0.0082	0.5586	0.077*	
C34	1.10601 (17)	0.14888 (19)	0.60772 (10)	0.0590 (5)	
H34	1.1764	0.1744	0.5928	0.071*	
C35	1.05280 (17)	0.21050 (15)	0.65028 (10)	0.0525 (4)	
H35	1.0875	0.2774	0.6642	0.063*	
C36	0.94712 (15)	0.17263 (13)	0.67245 (8)	0.0423 (3)	
H36	0.9113	0.2145	0.7011	0.051*	
N1	0.71034 (11)	0.33856 (10)	0.70041 (6)	0.0347 (3)	
N2	0.58371 (12)	0.37874 (10)	0.57026 (6)	0.0375 (3)	
N3	0.53706 (11)	0.17117 (11)	0.74359 (6)	0.0367 (3)	
O1	0.74771 (12)	−0.06575 (10)	0.65798 (6)	0.0535 (3)	
Fe1	0.82624 (2)	0.106179 (18)	0.901638 (10)	0.04051 (8)	

### Atomic displacement parameters (Å^2^)

**Table:**

	*U*^11^	*U*^22^	*U*^33^	*U*^12^	*U*^13^	*U*^23^
C1	0.0384 (8)	0.0414 (9)	0.0436 (8)	−0.0029 (6)	0.0034 (6)	0.0010 (7)
C2	0.0429 (8)	0.0394 (9)	0.0605 (10)	−0.0091 (7)	0.0063 (7)	−0.0014 (7)
C3	0.0542 (10)	0.0364 (8)	0.0620 (11)	−0.0066 (7)	0.0072 (8)	0.0125 (8)
C4	0.0540 (9)	0.0402 (9)	0.0468 (9)	−0.0024 (7)	0.0050 (7)	0.0129 (7)
C5	0.0361 (7)	0.0335 (7)	0.0362 (7)	0.0010 (6)	0.0080 (5)	0.0042 (6)
C6	0.0311 (6)	0.0316 (7)	0.0378 (7)	0.0020 (5)	0.0087 (5)	0.0024 (6)
C7	0.0326 (6)	0.0308 (7)	0.0308 (6)	0.0025 (5)	0.0056 (5)	0.0039 (5)
C8	0.0331 (6)	0.0331 (7)	0.0303 (6)	0.0026 (5)	0.0051 (5)	0.0030 (5)
C9	0.0340 (7)	0.0351 (7)	0.0335 (7)	0.0011 (6)	0.0033 (5)	0.0011 (6)
C10	0.0325 (6)	0.0336 (7)	0.0346 (7)	0.0001 (5)	0.0034 (5)	0.0013 (5)
C11	0.0329 (6)	0.0303 (7)	0.0304 (6)	0.0001 (5)	0.0041 (5)	0.0044 (5)
C12	0.0428 (8)	0.0370 (8)	0.0453 (8)	−0.0047 (6)	0.0043 (6)	0.0039 (6)
C13	0.0417 (8)	0.0406 (9)	0.0533 (9)	−0.0076 (7)	0.0032 (7)	−0.0079 (7)
C14	0.0421 (8)	0.0514 (10)	0.0431 (8)	−0.0023 (7)	−0.0025 (6)	−0.0073 (7)
C15	0.0453 (8)	0.0433 (9)	0.0351 (7)	0.0004 (7)	−0.0018 (6)	0.0022 (6)
C16	0.0309 (6)	0.0296 (7)	0.0305 (6)	−0.0013 (5)	0.0037 (5)	0.0047 (5)
C17	0.0368 (7)	0.0309 (7)	0.0317 (6)	−0.0032 (5)	0.0041 (5)	0.0059 (5)
C18	0.0364 (7)	0.0469 (9)	0.0416 (8)	−0.0014 (6)	0.0097 (6)	0.0133 (7)
C19	0.0478 (9)	0.0547 (11)	0.0567 (10)	0.0158 (8)	0.0169 (8)	0.0109 (8)
C20	0.0423 (7)	0.0258 (7)	0.0328 (7)	0.0015 (5)	0.0042 (5)	0.0039 (5)
C21	0.0561 (9)	0.0358 (8)	0.0368 (7)	−0.0023 (7)	0.0055 (6)	0.0084 (6)
C22	0.0760 (12)	0.0375 (9)	0.0335 (7)	0.0053 (8)	−0.0028 (7)	0.0063 (6)
C23	0.0516 (9)	0.0430 (9)	0.0476 (9)	0.0111 (7)	−0.0103 (7)	−0.0004 (7)
C24	0.0416 (8)	0.0373 (8)	0.0402 (8)	0.0070 (6)	0.0022 (6)	0.0009 (6)
C25	0.0948 (16)	0.0362 (9)	0.0540 (11)	0.0184 (10)	−0.0106 (10)	−0.0076 (8)
C26	0.0904 (16)	0.0484 (11)	0.0713 (13)	0.0181 (11)	0.0213 (12)	−0.0116 (10)
C27	0.122 (2)	0.0503 (12)	0.0429 (10)	0.0134 (12)	−0.0008 (12)	−0.0095 (9)
C28	0.0866 (16)	0.0472 (12)	0.0813 (16)	−0.0079 (11)	−0.0144 (13)	−0.0178 (11)
C29	0.116 (2)	0.0349 (10)	0.0681 (13)	−0.0096 (11)	0.0222 (13)	−0.0052 (9)
C30	0.0360 (7)	0.0339 (7)	0.0343 (7)	0.0012 (6)	0.0026 (5)	0.0026 (6)
C31	0.0330 (7)	0.0399 (8)	0.0340 (7)	0.0051 (6)	0.0042 (5)	0.0051 (6)
C32	0.0416 (8)	0.0631 (12)	0.0527 (9)	0.0003 (8)	0.0102 (7)	−0.0130 (8)
C33	0.0474 (10)	0.0894 (16)	0.0589 (11)	0.0020 (10)	0.0213 (8)	−0.0131 (11)
C34	0.0423 (9)	0.0791 (14)	0.0595 (11)	0.0001 (9)	0.0194 (8)	0.0125 (10)
C35	0.0469 (9)	0.0459 (10)	0.0655 (11)	−0.0037 (7)	0.0129 (8)	0.0134 (8)
C36	0.0423 (8)	0.0374 (8)	0.0488 (8)	0.0022 (6)	0.0127 (6)	0.0047 (7)
N1	0.0363 (6)	0.0336 (6)	0.0331 (6)	−0.0005 (5)	0.0037 (5)	0.0023 (5)
N2	0.0431 (7)	0.0343 (7)	0.0337 (6)	−0.0006 (5)	0.0037 (5)	0.0055 (5)
N3	0.0369 (6)	0.0386 (7)	0.0359 (6)	0.0032 (5)	0.0101 (5)	0.0066 (5)
O1	0.0592 (7)	0.0420 (7)	0.0625 (8)	−0.0106 (6)	0.0195 (6)	−0.0132 (6)
Fe1	0.05287 (15)	0.03087 (13)	0.03530 (13)	0.00303 (9)	0.00166 (9)	−0.00061 (8)

### Geometric parameters (Å, º)

**Table:**

C1—C2	1.368 (2)	C20—C21	1.421 (2)
C1—C6	1.405 (2)	C20—C24	1.424 (2)
C1—H1	0.9300	C20—Fe1	2.0393 (14)
C2—C3	1.395 (3)	C21—C22	1.425 (2)
C2—H2	0.9300	C21—Fe1	2.0454 (17)
C3—C4	1.362 (3)	C21—H21	0.9300
C3—H3	0.9300	C22—C23	1.403 (3)
C4—C5	1.410 (2)	C22—Fe1	2.0398 (17)
C4—H4	0.9300	C22—H22	0.9300
C5—N2	1.3727 (19)	C23—C24	1.423 (2)
C5—C6	1.419 (2)	C23—Fe1	2.0430 (17)
C6—N1	1.3777 (19)	C23—H23	0.9300
C7—N1	1.2975 (18)	C24—Fe1	2.0409 (16)
C7—C8	1.4241 (18)	C24—H24	0.9300
C7—C11	1.5244 (19)	C25—C29	1.397 (4)
C8—N2	1.3081 (18)	C25—C26	1.413 (3)
C8—C9	1.459 (2)	C25—Fe1	2.0320 (19)
C9—C15	1.3839 (19)	C25—H25	0.9300
C9—C10	1.397 (2)	C26—C27	1.398 (3)
C10—C12	1.389 (2)	C26—Fe1	2.031 (2)
C10—C11	1.5306 (19)	C26—H26	0.9300
C11—N3	1.4724 (18)	C27—C28	1.388 (4)
C11—C16	1.5531 (18)	C27—Fe1	2.039 (2)
C12—C13	1.388 (2)	C27—H27	0.9300
C12—H12	0.9300	C28—C29	1.404 (3)
C13—C14	1.379 (3)	C28—Fe1	2.041 (2)
C13—H13	0.9300	C28—H28	0.9300
C14—C15	1.380 (2)	C29—Fe1	2.035 (2)
C14—H14	0.9300	C29—H29	0.9300
C15—H15	0.9300	C30—O1	1.2141 (19)
C16—C30	1.523 (2)	C30—C31	1.498 (2)
C16—C17	1.5428 (19)	C31—C36	1.390 (2)
C16—H16	0.9800	C31—C32	1.397 (2)
C17—C20	1.5031 (19)	C32—C33	1.383 (3)
C17—C18	1.537 (2)	C32—H32	0.9300
C17—H17	0.9800	C33—C34	1.376 (3)
C18—N3	1.458 (2)	C33—H33	0.9300
C18—H18A	0.9700	C34—C35	1.377 (3)
C18—H18B	0.9700	C34—H34	0.9300
C19—N3	1.455 (2)	C35—C36	1.391 (2)
C19—H19A	0.9600	C35—H35	0.9300
C19—H19B	0.9600	C36—H36	0.9300
C19—H19C	0.9600		
			
C2—C1—C6	120.49 (15)	C29—C25—C26	107.95 (19)
C2—C1—H1	119.8	C29—C25—Fe1	70.05 (12)
C6—C1—H1	119.8	C26—C25—Fe1	69.63 (11)
C1—C2—C3	120.38 (16)	C29—C25—H25	126.0
C1—C2—H2	119.8	C26—C25—H25	126.0
C3—C2—H2	119.8	Fe1—C25—H25	125.9
C4—C3—C2	120.66 (16)	C27—C26—C25	107.3 (2)
C4—C3—H3	119.7	C27—C26—Fe1	70.21 (13)
C2—C3—H3	119.7	C25—C26—Fe1	69.68 (12)
C3—C4—C5	120.61 (16)	C27—C26—H26	126.3
C3—C4—H4	119.7	C25—C26—H26	126.3
C5—C4—H4	119.7	Fe1—C26—H26	125.4
N2—C5—C4	119.40 (13)	C28—C27—C26	108.7 (2)
N2—C5—C6	121.87 (13)	C28—C27—Fe1	70.20 (12)
C4—C5—C6	118.71 (14)	C26—C27—Fe1	69.62 (12)
N1—C6—C1	119.36 (13)	C28—C27—H27	125.6
N1—C6—C5	121.47 (13)	C26—C27—H27	125.6
C1—C6—C5	119.14 (14)	Fe1—C27—H27	126.1
N1—C7—C8	123.72 (13)	C27—C28—C29	108.1 (2)
N1—C7—C11	125.80 (12)	C27—C28—Fe1	70.03 (13)
C8—C7—C11	110.45 (12)	C29—C28—Fe1	69.63 (12)
N2—C8—C7	123.33 (13)	C27—C28—H28	126.0
N2—C8—C9	128.60 (12)	C29—C28—H28	126.0
C7—C8—C9	108.02 (12)	Fe1—C28—H28	126.0
C15—C9—C10	121.37 (14)	C25—C29—C28	107.9 (2)
C15—C9—C8	129.24 (14)	C25—C29—Fe1	69.78 (12)
C10—C9—C8	109.37 (12)	C28—C29—Fe1	70.08 (13)
C12—C10—C9	119.54 (13)	C25—C29—H29	126.0
C12—C10—C11	129.59 (13)	C28—C29—H29	126.0
C9—C10—C11	110.67 (12)	Fe1—C29—H29	125.7
N3—C11—C7	108.92 (12)	O1—C30—C31	119.77 (14)
N3—C11—C10	114.60 (12)	O1—C30—C16	120.18 (14)
C7—C11—C10	101.34 (11)	C31—C30—C16	120.04 (13)
N3—C11—C16	100.40 (10)	C36—C31—C32	118.61 (15)
C7—C11—C16	113.68 (11)	C36—C31—C30	123.70 (14)
C10—C11—C16	118.13 (12)	C32—C31—C30	117.66 (15)
C10—C12—C13	118.70 (15)	C33—C32—C31	120.51 (18)
C10—C12—H12	120.6	C33—C32—H32	119.7
C13—C12—H12	120.6	C31—C32—H32	119.7
C14—C13—C12	121.21 (15)	C34—C33—C32	120.18 (18)
C14—C13—H13	119.4	C34—C33—H33	119.9
C12—C13—H13	119.4	C32—C33—H33	119.9
C15—C14—C13	120.64 (15)	C33—C34—C35	120.27 (17)
C15—C14—H14	119.7	C33—C34—H34	119.9
C13—C14—H14	119.7	C35—C34—H34	119.9
C14—C15—C9	118.53 (15)	C34—C35—C36	119.95 (18)
C14—C15—H15	120.7	C34—C35—H35	120.0
C9—C15—H15	120.7	C36—C35—H35	120.0
C30—C16—C17	112.41 (12)	C31—C36—C35	120.48 (16)
C30—C16—C11	115.39 (11)	C31—C36—H36	119.8
C17—C16—C11	104.26 (11)	C35—C36—H36	119.8
C30—C16—H16	108.2	C7—N1—C6	114.88 (12)
C17—C16—H16	108.2	C8—N2—C5	114.69 (12)
C11—C16—H16	108.2	C19—N3—C18	113.81 (13)
C20—C17—C18	115.40 (12)	C19—N3—C11	116.15 (12)
C20—C17—C16	111.96 (11)	C18—N3—C11	107.73 (12)
C18—C17—C16	104.99 (11)	C25—Fe1—C26	40.69 (9)
C20—C17—H17	108.1	C25—Fe1—C29	40.17 (10)
C18—C17—H17	108.1	C26—Fe1—C29	67.93 (11)
C16—C17—H17	108.1	C25—Fe1—C20	107.37 (7)
N3—C18—C17	105.08 (11)	C26—Fe1—C20	123.10 (9)
N3—C18—H18A	110.7	C29—Fe1—C20	122.47 (8)
C17—C18—H18A	110.7	C25—Fe1—C22	157.98 (10)
N3—C18—H18B	110.7	C26—Fe1—C22	122.02 (9)
C17—C18—H18B	110.7	C29—Fe1—C22	160.21 (10)
H18A—C18—H18B	108.8	C20—Fe1—C22	68.65 (6)
N3—C19—H19A	109.5	C25—Fe1—C27	67.58 (8)
N3—C19—H19B	109.5	C26—Fe1—C27	40.17 (10)
H19A—C19—H19B	109.5	C29—Fe1—C27	67.37 (10)
N3—C19—H19C	109.5	C20—Fe1—C27	159.57 (10)
H19A—C19—H19C	109.5	C22—Fe1—C27	108.15 (8)
H19B—C19—H19C	109.5	C25—Fe1—C28	67.55 (10)
C21—C20—C24	107.48 (13)	C26—Fe1—C28	67.55 (11)
C21—C20—C17	126.70 (14)	C29—Fe1—C28	40.28 (10)
C24—C20—C17	125.70 (13)	C20—Fe1—C28	158.62 (10)
C21—C20—Fe1	69.88 (9)	C22—Fe1—C28	123.98 (8)
C24—C20—Fe1	69.64 (8)	C27—Fe1—C28	39.77 (11)
C17—C20—Fe1	128.88 (10)	C25—Fe1—C23	160.48 (9)
C20—C21—C22	107.87 (15)	C26—Fe1—C23	157.49 (9)
C20—C21—Fe1	69.41 (9)	C29—Fe1—C23	124.55 (10)
C22—C21—Fe1	69.38 (10)	C20—Fe1—C23	68.77 (6)
C20—C21—H21	126.1	C22—Fe1—C23	40.19 (8)
C22—C21—H21	126.1	C27—Fe1—C23	122.67 (8)
Fe1—C21—H21	126.7	C28—Fe1—C23	108.65 (9)
C23—C22—C21	108.49 (14)	C25—Fe1—C24	123.92 (8)
C23—C22—Fe1	70.03 (10)	C26—Fe1—C24	160.03 (8)
C21—C22—Fe1	69.80 (9)	C29—Fe1—C24	108.51 (9)
C23—C22—H22	125.8	C20—Fe1—C24	40.84 (6)
C21—C22—H22	125.8	C22—Fe1—C24	68.12 (7)
Fe1—C22—H22	126.0	C27—Fe1—C24	158.44 (9)
C22—C23—C24	107.97 (15)	C28—Fe1—C24	123.35 (10)
C22—C23—Fe1	69.78 (10)	C23—Fe1—C24	40.78 (6)
C24—C23—Fe1	69.53 (9)	C25—Fe1—C21	122.09 (9)
C22—C23—H23	126.0	C26—Fe1—C21	107.18 (9)
C24—C23—H23	126.0	C29—Fe1—C21	157.89 (9)
Fe1—C23—H23	126.2	C20—Fe1—C21	40.71 (6)
C20—C24—C23	108.19 (15)	C22—Fe1—C21	40.82 (7)
C20—C24—Fe1	69.52 (9)	C27—Fe1—C21	123.73 (10)
C23—C24—Fe1	69.69 (10)	C28—Fe1—C21	159.79 (9)
C20—C24—H24	125.9	C23—Fe1—C21	68.28 (8)
C23—C24—H24	125.9	C24—Fe1—C21	68.29 (7)
Fe1—C24—H24	126.5		
			
C6—C1—C2—C3	0.1 (3)	C25—C26—Fe1—C22	−161.76 (12)
C1—C2—C3—C4	−0.5 (3)	C25—C26—Fe1—C27	118.1 (2)
C2—C3—C4—C5	0.1 (3)	C27—C26—Fe1—C28	−36.90 (15)
C3—C4—C5—N2	179.13 (16)	C25—C26—Fe1—C28	81.19 (15)
C3—C4—C5—C6	0.6 (3)	C27—C26—Fe1—C23	47.7 (3)
C2—C1—C6—N1	−177.54 (15)	C25—C26—Fe1—C23	165.8 (2)
C2—C1—C6—C5	0.6 (2)	C27—C26—Fe1—C24	−164.1 (2)
N2—C5—C6—N1	−1.4 (2)	C25—C26—Fe1—C24	−46.0 (3)
C4—C5—C6—N1	177.16 (14)	C27—C26—Fe1—C21	122.34 (15)
N2—C5—C6—C1	−179.44 (14)	C25—C26—Fe1—C21	−119.57 (13)
C4—C5—C6—C1	−0.9 (2)	C28—C29—Fe1—C25	118.8 (2)
N1—C7—C8—N2	−2.5 (2)	C25—C29—Fe1—C26	−37.98 (13)
C11—C7—C8—N2	175.52 (13)	C28—C29—Fe1—C26	80.85 (17)
N1—C7—C8—C9	179.88 (13)	C25—C29—Fe1—C20	78.16 (14)
C11—C7—C8—C9	−2.13 (16)	C28—C29—Fe1—C20	−163.01 (15)
N2—C8—C9—C15	0.7 (3)	C25—C29—Fe1—C22	−162.3 (2)
C7—C8—C9—C15	178.24 (15)	C28—C29—Fe1—C22	−43.4 (3)
N2—C8—C9—C10	−177.84 (15)	C25—C29—Fe1—C27	−81.57 (15)
C7—C8—C9—C10	−0.34 (16)	C28—C29—Fe1—C27	37.26 (16)
C15—C9—C10—C12	−0.7 (2)	C25—C29—Fe1—C28	−118.8 (2)
C8—C9—C10—C12	178.05 (14)	C25—C29—Fe1—C23	163.40 (11)
C15—C9—C10—C11	−176.03 (14)	C28—C29—Fe1—C23	−77.77 (18)
C8—C9—C10—C11	2.67 (17)	C25—C29—Fe1—C24	121.06 (12)
N1—C7—C11—N3	60.24 (18)	C28—C29—Fe1—C24	−120.12 (16)
C8—C7—C11—N3	−117.69 (12)	C25—C29—Fe1—C21	43.3 (3)
N1—C7—C11—C10	−178.59 (14)	C28—C29—Fe1—C21	162.2 (2)
C8—C7—C11—C10	3.48 (15)	C21—C20—Fe1—C25	−119.34 (12)
N1—C7—C11—C16	−50.78 (19)	C24—C20—Fe1—C25	122.16 (11)
C8—C7—C11—C16	131.29 (12)	C17—C20—Fe1—C25	2.14 (16)
C12—C10—C11—N3	−61.4 (2)	C21—C20—Fe1—C26	−77.46 (13)
C9—C10—C11—N3	113.40 (14)	C24—C20—Fe1—C26	164.04 (11)
C12—C10—C11—C7	−178.49 (15)	C17—C20—Fe1—C26	44.02 (17)
C9—C10—C11—C7	−3.70 (15)	C21—C20—Fe1—C29	−160.75 (12)
C12—C10—C11—C16	56.6 (2)	C24—C20—Fe1—C29	80.75 (13)
C9—C10—C11—C16	−128.57 (13)	C17—C20—Fe1—C29	−39.27 (18)
C9—C10—C12—C13	−0.4 (2)	C21—C20—Fe1—C22	37.68 (10)
C11—C10—C12—C13	174.02 (15)	C24—C20—Fe1—C22	−80.82 (10)
C10—C12—C13—C14	1.0 (3)	C17—C20—Fe1—C22	159.16 (16)
C12—C13—C14—C15	−0.6 (3)	C21—C20—Fe1—C27	−47.1 (2)
C13—C14—C15—C9	−0.5 (3)	C24—C20—Fe1—C27	−165.6 (2)
C10—C9—C15—C14	1.1 (2)	C17—C20—Fe1—C27	74.4 (3)
C8—C9—C15—C14	−177.37 (15)	C21—C20—Fe1—C28	168.0 (2)
N3—C11—C16—C30	159.56 (12)	C24—C20—Fe1—C28	49.5 (2)
C7—C11—C16—C30	−84.31 (15)	C17—C20—Fe1—C28	−70.5 (3)
C10—C11—C16—C30	34.24 (17)	C21—C20—Fe1—C23	80.96 (11)
N3—C11—C16—C17	35.78 (13)	C24—C20—Fe1—C23	−37.54 (10)
C7—C11—C16—C17	151.92 (11)	C17—C20—Fe1—C23	−157.56 (16)
C10—C11—C16—C17	−89.54 (14)	C21—C20—Fe1—C24	118.50 (13)
C30—C16—C17—C20	90.90 (14)	C17—C20—Fe1—C24	−120.02 (17)
C11—C16—C17—C20	−143.42 (12)	C24—C20—Fe1—C21	−118.50 (13)
C30—C16—C17—C18	−143.18 (12)	C17—C20—Fe1—C21	121.48 (18)
C11—C16—C17—C18	−17.50 (14)	C23—C22—Fe1—C25	165.56 (18)
C20—C17—C18—N3	115.98 (13)	C21—C22—Fe1—C25	46.0 (2)
C16—C17—C18—N3	−7.78 (15)	C23—C22—Fe1—C26	−161.47 (12)
C18—C17—C20—C21	40.0 (2)	C21—C22—Fe1—C26	78.98 (13)
C16—C17—C20—C21	160.02 (14)	C23—C22—Fe1—C29	−46.0 (3)
C18—C17—C20—C24	−144.52 (15)	C21—C22—Fe1—C29	−165.6 (2)
C16—C17—C20—C24	−24.5 (2)	C23—C22—Fe1—C20	81.97 (10)
C18—C17—C20—Fe1	−52.85 (18)	C21—C22—Fe1—C20	−37.58 (10)
C16—C17—C20—Fe1	67.16 (17)	C23—C22—Fe1—C27	−119.49 (13)
C24—C20—C21—C22	0.84 (18)	C21—C22—Fe1—C27	120.96 (13)
C17—C20—C21—C22	176.98 (14)	C23—C22—Fe1—C28	−78.46 (15)
Fe1—C20—C21—C22	−58.91 (12)	C21—C22—Fe1—C28	161.99 (13)
C24—C20—C21—Fe1	59.75 (10)	C21—C22—Fe1—C23	−119.55 (14)
C17—C20—C21—Fe1	−124.11 (15)	C23—C22—Fe1—C24	37.89 (9)
C20—C21—C22—C23	−0.6 (2)	C21—C22—Fe1—C24	−81.66 (11)
Fe1—C21—C22—C23	−59.55 (13)	C23—C22—Fe1—C21	119.55 (14)
C20—C21—C22—Fe1	58.93 (11)	C28—C27—Fe1—C25	−81.37 (16)
C21—C22—C23—C24	0.2 (2)	C26—C27—Fe1—C25	38.47 (15)
Fe1—C22—C23—C24	−59.26 (12)	C28—C27—Fe1—C26	−119.8 (2)
C21—C22—C23—Fe1	59.41 (12)	C28—C27—Fe1—C29	−37.72 (15)
C21—C20—C24—C23	−0.75 (18)	C26—C27—Fe1—C29	82.12 (16)
C17—C20—C24—C23	−176.94 (14)	C28—C27—Fe1—C20	−160.86 (19)
Fe1—C20—C24—C23	59.15 (11)	C26—C27—Fe1—C20	−41.0 (3)
C21—C20—C24—Fe1	−59.90 (11)	C28—C27—Fe1—C22	121.69 (14)
C17—C20—C24—Fe1	123.91 (14)	C26—C27—Fe1—C22	−118.47 (15)
C22—C23—C24—C20	0.37 (19)	C26—C27—Fe1—C28	119.8 (2)
Fe1—C23—C24—C20	−59.04 (11)	C28—C27—Fe1—C23	79.83 (16)
C22—C23—C24—Fe1	59.41 (12)	C26—C27—Fe1—C23	−160.33 (14)
C29—C25—C26—C27	0.6 (2)	C28—C27—Fe1—C24	45.4 (3)
Fe1—C25—C26—C27	60.41 (14)	C26—C27—Fe1—C24	165.26 (19)
C29—C25—C26—Fe1	−59.81 (14)	C28—C27—Fe1—C21	164.07 (13)
C25—C26—C27—C28	−0.6 (2)	C26—C27—Fe1—C21	−76.09 (16)
Fe1—C26—C27—C28	59.50 (16)	C27—C28—Fe1—C25	81.45 (15)
C25—C26—C27—Fe1	−60.07 (14)	C29—C28—Fe1—C25	−37.69 (15)
C26—C27—C28—C29	0.3 (2)	C27—C28—Fe1—C26	37.26 (14)
Fe1—C27—C28—C29	59.46 (15)	C29—C28—Fe1—C26	−81.88 (17)
C26—C27—C28—Fe1	−59.15 (15)	C27—C28—Fe1—C29	119.1 (2)
C26—C25—C29—C28	−0.4 (2)	C27—C28—Fe1—C20	161.70 (18)
Fe1—C25—C29—C28	−59.96 (15)	C29—C28—Fe1—C20	42.6 (3)
C26—C25—C29—Fe1	59.55 (14)	C27—C28—Fe1—C22	−77.16 (16)
C27—C28—C29—C25	0.1 (2)	C29—C28—Fe1—C22	163.70 (15)
Fe1—C28—C29—C25	59.77 (15)	C29—C28—Fe1—C27	−119.1 (2)
C27—C28—C29—Fe1	−59.71 (16)	C27—C28—Fe1—C23	−119.02 (14)
C17—C16—C30—O1	31.91 (18)	C29—C28—Fe1—C23	121.84 (16)
C11—C16—C30—O1	−87.47 (17)	C27—C28—Fe1—C24	−161.74 (12)
C17—C16—C30—C31	−146.91 (12)	C29—C28—Fe1—C24	79.11 (17)
C11—C16—C30—C31	93.71 (15)	C27—C28—Fe1—C21	−41.4 (3)
O1—C30—C31—C36	−175.78 (15)	C29—C28—Fe1—C21	−160.5 (2)
C16—C30—C31—C36	3.0 (2)	C22—C23—Fe1—C25	−163.8 (2)
O1—C30—C31—C32	2.3 (2)	C24—C23—Fe1—C25	−44.5 (3)
C16—C30—C31—C32	−178.92 (13)	C22—C23—Fe1—C26	44.8 (3)
C36—C31—C32—C33	0.2 (3)	C24—C23—Fe1—C26	164.0 (2)
C30—C31—C32—C33	−177.96 (17)	C22—C23—Fe1—C29	162.79 (11)
C31—C32—C33—C34	−0.3 (3)	C24—C23—Fe1—C29	−77.98 (13)
C32—C33—C34—C35	0.4 (3)	C22—C23—Fe1—C20	−81.63 (10)
C33—C34—C35—C36	−0.3 (3)	C24—C23—Fe1—C20	37.60 (10)
C32—C31—C36—C35	−0.1 (2)	C24—C23—Fe1—C22	119.23 (15)
C30—C31—C36—C35	177.97 (14)	C22—C23—Fe1—C27	79.31 (14)
C34—C35—C36—C31	0.1 (3)	C24—C23—Fe1—C27	−161.46 (13)
C8—C7—N1—C6	1.1 (2)	C22—C23—Fe1—C28	120.96 (13)
C11—C7—N1—C6	−176.57 (13)	C24—C23—Fe1—C28	−119.81 (13)
C1—C6—N1—C7	178.75 (13)	C22—C23—Fe1—C24	−119.23 (15)
C5—C6—N1—C7	0.7 (2)	C22—C23—Fe1—C21	−37.74 (10)
C7—C8—N2—C5	1.7 (2)	C24—C23—Fe1—C21	81.49 (11)
C9—C8—N2—C5	178.80 (14)	C20—C24—Fe1—C25	−76.82 (13)
C4—C5—N2—C8	−178.38 (15)	C23—C24—Fe1—C25	163.60 (13)
C6—C5—N2—C8	0.1 (2)	C20—C24—Fe1—C26	−42.4 (3)
C17—C18—N3—C19	162.64 (13)	C23—C24—Fe1—C26	−162.0 (2)
C17—C18—N3—C11	32.35 (15)	C20—C24—Fe1—C29	−118.58 (11)
C7—C11—N3—C19	68.72 (17)	C23—C24—Fe1—C29	121.84 (13)
C10—C11—N3—C19	−43.96 (18)	C23—C24—Fe1—C20	−119.58 (14)
C16—C11—N3—C19	−171.64 (14)	C20—C24—Fe1—C22	82.22 (10)
C7—C11—N3—C18	−162.30 (11)	C23—C24—Fe1—C22	−37.36 (11)
C10—C11—N3—C18	85.02 (14)	C20—C24—Fe1—C27	166.3 (2)
C16—C11—N3—C18	−42.66 (14)	C23—C24—Fe1—C27	46.7 (3)
C29—C25—Fe1—C26	118.98 (19)	C20—C24—Fe1—C28	−160.61 (11)
C26—C25—Fe1—C29	−118.98 (19)	C23—C24—Fe1—C28	79.81 (14)
C29—C25—Fe1—C20	−120.09 (13)	C20—C24—Fe1—C23	119.58 (14)
C26—C25—Fe1—C20	120.93 (14)	C20—C24—Fe1—C21	38.10 (8)
C29—C25—Fe1—C22	164.03 (18)	C23—C24—Fe1—C21	−81.48 (11)
C26—C25—Fe1—C22	45.1 (3)	C20—C21—Fe1—C25	79.13 (12)
C29—C25—Fe1—C27	80.98 (15)	C22—C21—Fe1—C25	−161.43 (12)
C26—C25—Fe1—C27	−37.99 (15)	C20—C21—Fe1—C26	121.14 (11)
C29—C25—Fe1—C28	37.80 (14)	C22—C21—Fe1—C26	−119.42 (12)
C26—C25—Fe1—C28	−81.18 (16)	C20—C21—Fe1—C29	47.6 (3)
C29—C25—Fe1—C23	−44.7 (3)	C22—C21—Fe1—C29	167.1 (2)
C26—C25—Fe1—C23	−163.7 (2)	C22—C21—Fe1—C20	119.44 (15)
C29—C25—Fe1—C24	−78.24 (14)	C20—C21—Fe1—C22	−119.44 (15)
C26—C25—Fe1—C24	162.78 (13)	C20—C21—Fe1—C27	162.09 (11)
C29—C25—Fe1—C21	−162.25 (12)	C22—C21—Fe1—C27	−78.47 (14)
C26—C25—Fe1—C21	78.77 (15)	C20—C21—Fe1—C28	−167.4 (2)
C27—C26—Fe1—C25	−118.1 (2)	C22—C21—Fe1—C28	−47.9 (3)
C27—C26—Fe1—C29	−80.59 (16)	C20—C21—Fe1—C23	−82.26 (10)
C25—C26—Fe1—C29	37.51 (13)	C22—C21—Fe1—C23	37.18 (11)
C27—C26—Fe1—C20	164.13 (13)	C20—C21—Fe1—C24	−38.22 (9)
C25—C26—Fe1—C20	−77.78 (15)	C22—C21—Fe1—C24	81.23 (11)
C27—C26—Fe1—C22	80.15 (17)		

### Hydrogen-bond geometry (Å, º)

Cg3 is the centroid of the C20–C24 ring.

**Table:**

*D*—H···*A*	*D*—H	H···*A*	*D*···*A*	*D*—H···*A*
C25—H25···N3	0.93	2.55	3.351 (2)	144
C35—H35···*Cg*3^i^	0.93	2.83	3.616 (2)	143

Symmetry code: (i) −*x*+2, *y*+1/2, −*z*+3/2.
